# Supplementary material for: Simulated Fermentation of Strong-Flavor Baijiu through Functional Microbial Combination to Realize the Stable Synthesis of Important Flavor Chemicals
Source: Foods. 2023 Feb 2;12(3):644. doi: 10.3390/foods12030644 (PMC9913964; doi:10.3390/foods12030644)
Supplement: Supplementary file 1 [file foods-12-00644-s001.zip › foods-2154514-supplementary.pdf]

Supplementary

Simulated Fermentation of Strong-Flavor Baijiu through Functional Microbial Combination to Realize the Stable Synthesis of Important Flavor Chemicals

Youqiang Xu [1,*,†], Mengqin Wu [1,†], Dong Zhao [2], Jia Zheng [2], Mengqi Dai [1], Xiuting Li [3,4,*], Weiwei Li [1], Chengnan Zhang [1] and Baoguo Sun [3,4]

[1] School of Food and Health, Beijing Technology and Business University (BTBU), Beijing 100048, China
[2] Wuliangye Yibin Co., Ltd. Yibin 644000, China
[3] Key Laboratory of Brewing Microbiome and Enzymatic Molecular Engineering, China General Chamber of Commerce, Beijing 102401, China
[4] Key Laboratory of Brewing Molecular Engineering of China Light Industry, Beijing Technology and Business University, Beijing 100048, China
* Correspondence: xuyouqiang@btbu.edu.cn (Y.X.); lixt@btbu.edu.cn (X.L.)
† These authors contributed equally to this work.

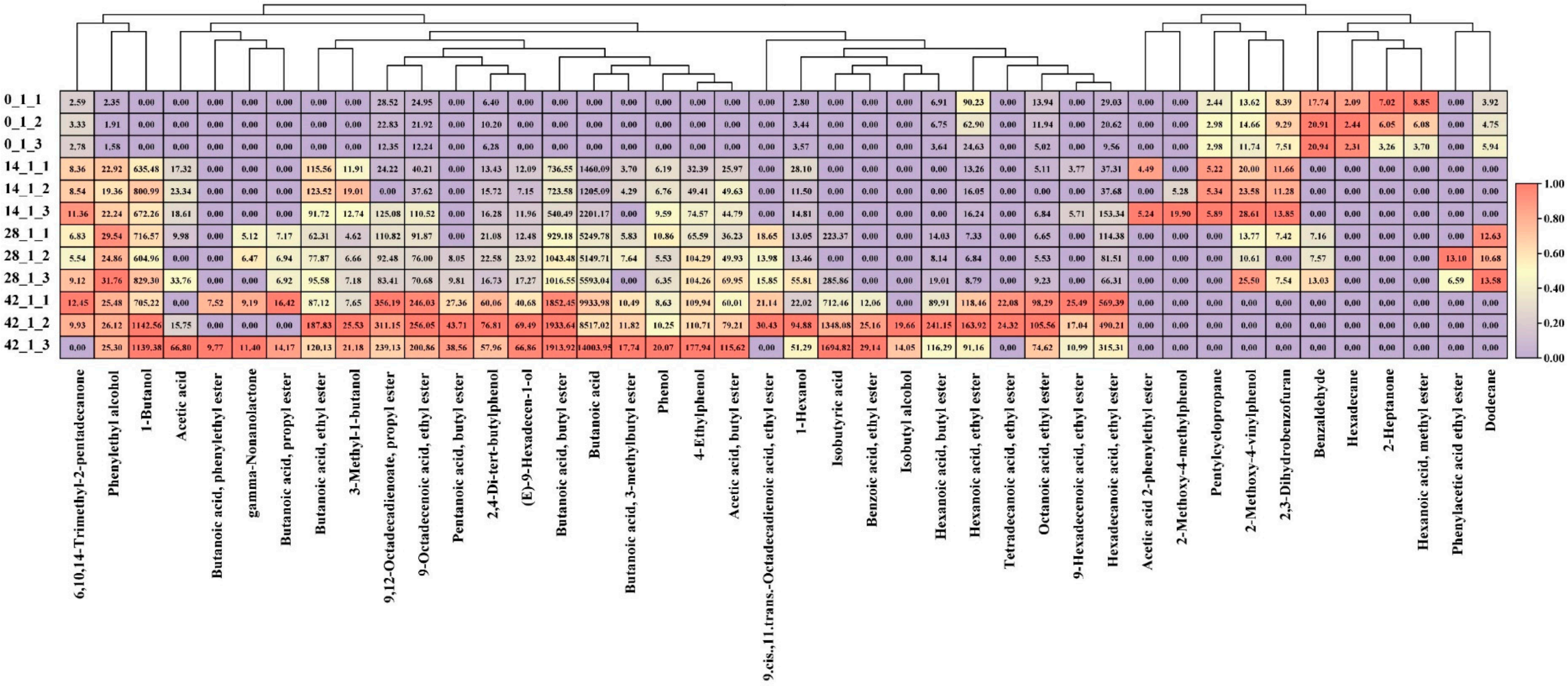

Figure S1. Heatmap analysis of flavor compounds in fermented grains of sample 1.

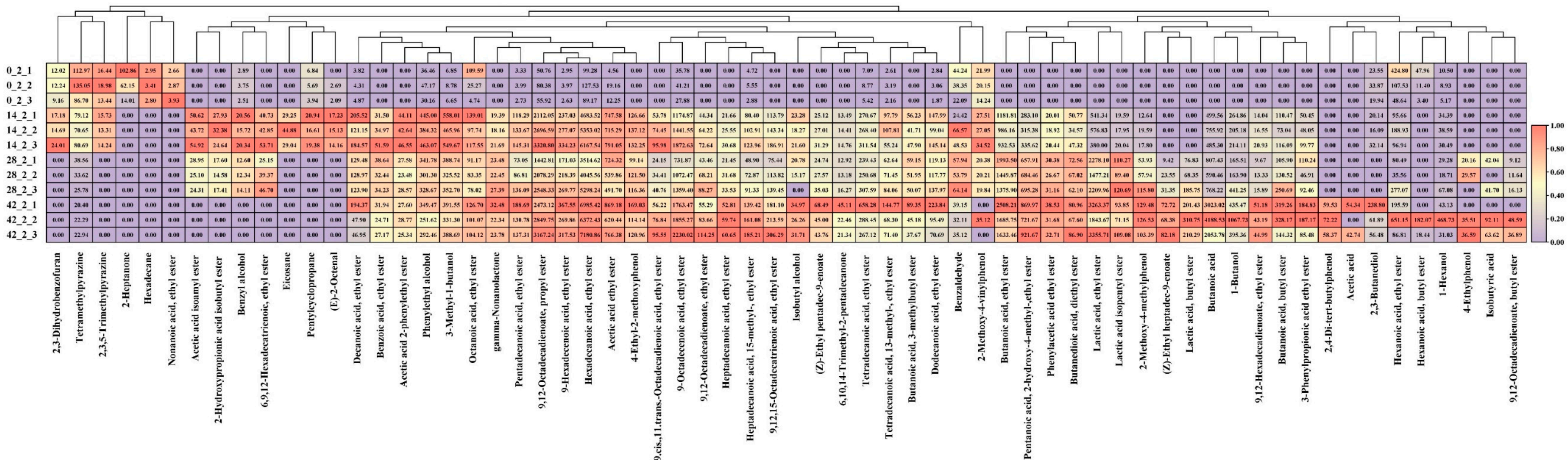

Figure S2. Heatmap analysis of flavor compounds in fermented grains of sample 2.

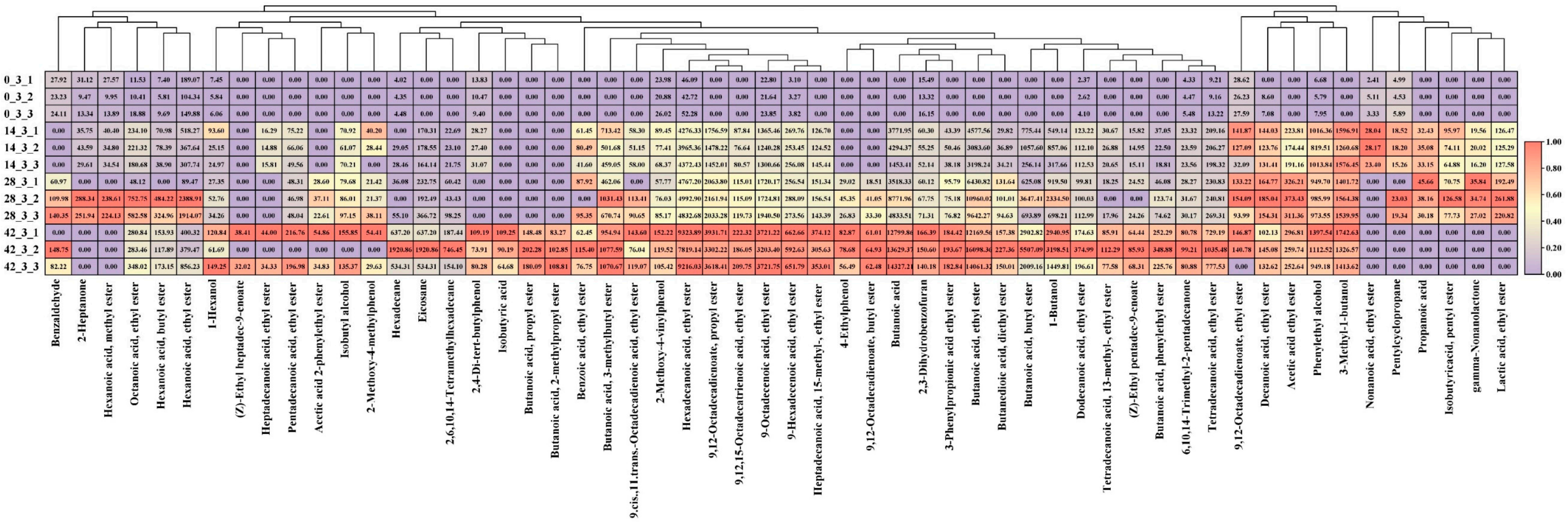

Figure S3. Heatmap analysis of flavor compounds in fermented grains of sample 3.

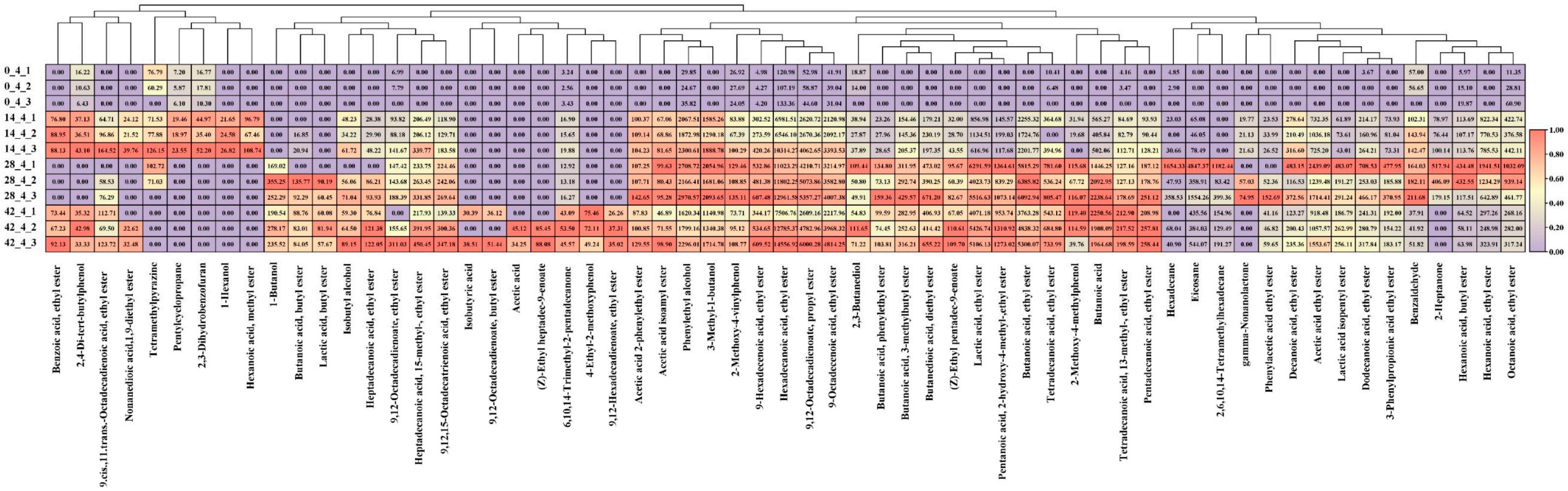

Figure S4. Heatmap analysis of flavor compounds in fermented grains of sample 4.

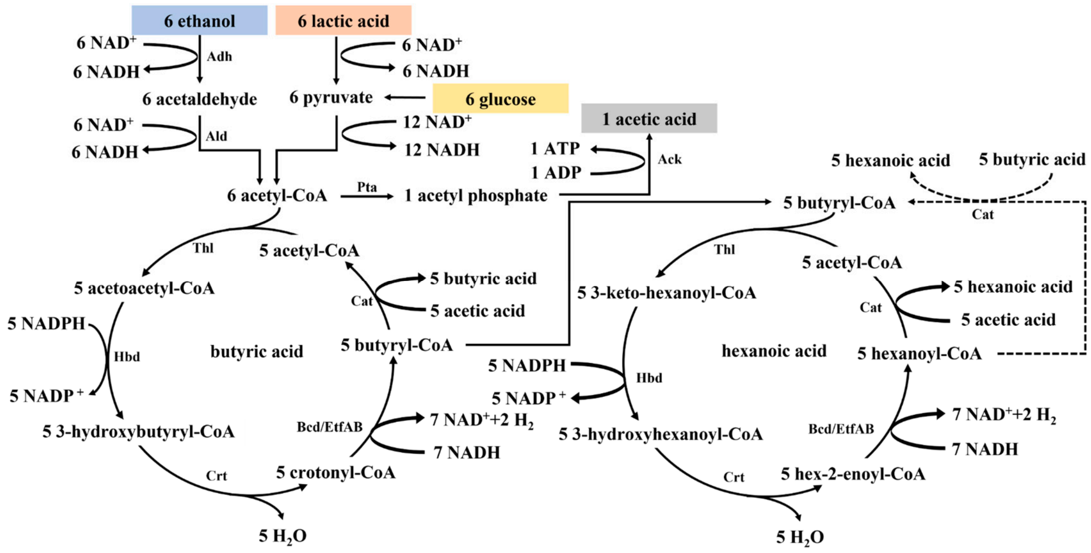

Figure S5. Synthetic pathways of fatty acids acetic acid, butyric acid and hexanoic acid.

Table S1. Relative concentrations of flavor compounds of all samples during fermentation (μg/kg).

Flavor compounds [a]	1_0	1_14	1_28	1_42	2_0	2_14	2_28	2_42	3_0	3_14	3_28	3_42	4_0	4_14	4_28	4_42
Phenylethyl alcohol	1.95±0.39	21.51±1.89	28.72±3.52	25.63±0.43	37.93±8.60	430.80±41.25	323.91±20.66	297.85±49.15	6.81±1.08	949.90±112.93	969.75±18.44	1153.08±226.91	30.11±5.58	2080.37±214.10	2615.23±410.15	1905.17±350.09
Benzaldehyde	19.86±1.84	ND	9.25±3.28	ND	34.89±11.47	46.51±21.15	58.62±5.21	35.46±3.53	25.08±2.49	ND	103.76±40.05	76.99±74.51	37.88±32.81	129.57±23.62	185.94±24.05	43.89±7.16
Butanoic acid	ND	1622.12±517.43	5330.85±232.52	10818.32±2848.36	ND	580.26±152.29	722.04±115.62	3088.44±1068.88	ND	3173.24±1512.15	5707.93±2733.79	13585.48±764.62	ND	491.06±80.28	1925.95±421.77	2041.11±183.58
Isobutyric acid	ND	ND	169.74±150.28	1251.79±498.21	ND	ND	27.91±24.17	51.91±47.16	ND	ND	ND	88.04±22.36	ND	ND	ND	22.97±20.30
2-Heptanone	5.45±1.96	ND	ND	ND	59.67±44.48	ND	ND	ND	17.98±11.55	36.32±7.01	180.09±157.02	ND	ND	85.18±13.02	367.73±172.62	ND
6,10,14-Trimethyl-2-pentadecanone	2.90±0.39	9.42±1.68	7.16±1.82	7.46±6.58	ND	14.22±0.65	14.12±1.86	29.64±13.41	4.76±0.63	23.49±0.15	30.04±1.70	86.96±10.61	3.08±0.46	17.47±2.17	14.12±1.86	47.39±5.44
Hexadecane	2.28±0.18	ND	ND	ND	3.05±0.32	ND	ND	ND	4.28±0.24	19.17±16.6	30.39±27.98	1030.79±772.53	2.59±2.44	17.89±15.96	686.93±852.07	36.31±34.25
Pentylcyclopropane	2.80±0.31	5.48±0.36	ND	ND	5.49±1.46	18.98±2.19	ND	ND	5.13±0.69	17.32±1.80	14.12±12.37	ND	6.39±0.71	20.66±2.51	ND	ND
9,12-Octadecadienoate, propyl ester	21.23±8.20	49.76±66.34	95.57±13.96	302.16±59.05	62.35±15.82	2709.82±604.48	2023.15±554.82	2830.04±347.48	ND	1562.27±168.79	2086.34±67.23	3617.45±314.75	52.15±7.17	3117.91±818.55	4880.61±597.21	4464.13±1717.90
9.cis.,11.trans.-Octadecadienoic acid, ethyl ester	ND	ND	16.16±2.35	17.19±15.60	ND	74.74±21.10	33.11±8.38	76.20±19.67	ND	55.82±4.05	68.02±60.00	112.90±34.20	ND	108.70±50.95	44.94±39.92	101.97±28.66
9-Hexadecenoic acid, ethyl ester	ND	3.16±2.90	ND	17.84±7.28	3.18±0.70	282.78±48.85	219.73±49.38	318.32±48.85	3.40±0.38	259.76±8.76	272.73±15.79	635.69±37.68	4.48±0.43	332.12±77.69	540.57±63.41	496.11±136.81
Isobutyl alcohol	ND	ND	ND	11.24±10.13	ND	21.05±2.55	11.98±10.75	30.98±4.40	ND	67.40±5.49	87.61±8.85	97.07±84.69	ND	48.06±13.75	42.37±37.45	70.98±15.95
gamma-Nonanolactone	ND	ND	3.86±3.41	6.86±6.04	ND	19.74±1.79	24.44±2.61	26.20±5.49	ND	18.59±2.09	32.53±4.80	ND	ND	20.84±0.96	43.99±39.14	ND
Benzoic acid, ethyl ester	ND	ND	ND	22.12±8.94	ND	39.36±10.74	35.10±3.19	27.94±3.67	ND	61.18±19.45	61.09±53.04	84.87±27.39	ND	84.63±6.79	ND	77.60±12.96
Butanoic acid, butyl ester	ND	666.87±109.65	996.41±59.75	1900.01±42.35	ND	99.87±23.40	162.37±77.47	263.92±103.67	ND	696.39±406.54	1655.46±1725.42	3473.03±1817.34	ND	12.6±11.10	76.02±69.33	85.27±3.07
Butanoic acid, ethyl ester	ND	110.27±16.55	78.58±16.64	131.70±51.34	ND	1033.50±131.21	1606.42±337.25	1942.47±490.64	ND	3619.80±831.42	9011.04±2329.65	14109.75±1964.85	ND	2060.62±292.09	6098.02±285.30	4633.89±788.53
Butanoic acid, 3-methylbutyl ester	ND	2.66±2.33	4.49±3.99	13.35±3.86	ND	48.61±7.28	53.72±4.79	57.40±27.92	ND	558.05±136.24	721.41±288.05	1034.40±68.90	ND	168.40±32.34	344.75±74.08	283.93±31.80
9-Octadecenoic acid, ethyl ester	19.70±6.64	62.79±41.36	79.51±11.02	234.31±29.40	34.95±6.70	1496.35±352.10	1054.58±314.15	1949.59±247.16	22.76±1.11	1302.13±62.60	1795.16±125.89	3548.79±299.11	37.33±5.63	2535.56±743.16	3601.72±396.54	3666.84±1324.14
Hexanoic acid, butyl	5.77±1.85	ND	13.73±	149.12±	20.92±	ND	ND	66.84±	7.63±1.95	62.75±	269.73±	148.32±	13.65±	111.54±	328.18±	62.20±

ester			5.44	80.79	23.75			100.22		20.99	246.79	28.06	7.06	3.79	182.44	3.56
Hexanoic acid, ethyl ester	59.25±32.95	15.18±1.67	7.65±1.01	124.51±36.76	193.65±202.33	127.18±53.48	131.04±128.45	311.18±299.40	147.77±42.40	397.88±108.47	1464.15±1213.95	545.34±269.44	ND	792.80±26.66	1272.90±650.17	290.05±37.99
Tetradecanoic acid, ethyl ester	ND	ND	ND	15.47±13.44	7.09±1.67	283.54±24.28	265.90±36.54	404.62±219.94	10.53±2.33	204.58±5.62	246.98±19.97	847.40±164.67	5.63±5.26	253.21±219.81	707.77±149.03	653.97±99.10
Octanoic acid, ethyl ester	10.30±4.68	3.99±3.56	7.14±1.89	92.82±16.18	46.53±55.56	118.10±20.64	84.18±6.61	110.63±14.00	13.61±4.60	212.04±27.90	461.15±367.67	304.11±38.05	33.69±25.13	413.81±33.66	811.00±305.99	289.13±25.31
3-Methyl-1-butanol	ND	14.55±3.88	6.15±1.36	18.12±9.33	7.43±1.18	524.55±50.91	355.65±31.71	370.51±33.99	ND	1478.01±188.49	1502.02±87.72	1494.27±219.44	ND	1588.07±299.31	1943.23±227.86	1398.71±291.31
Acetic acid 2-phenylethyl ester	ND	3.24±2.83	ND	ND	ND	44.44±1.98	26.54±2.70	27.23±1.74	ND	ND	29.44±7.29	29.90±27.76	ND	104.58±4.39	119.20±20.31	106.08±21.35
Hexadecanoic acid, ethyl ester	19.74±9.77	76.11±66.88	87.40±24.57	458.31±130.01	105.33±19.88	5401.36±743.19	4286.14±915.82	6846.23±421.80	47.03±4.85	4204.71±212.77	4864.26±116.12	8786.35±839.36	120.51±13.09	7947.29±2061.39	11929.04±975.34	11616.35±3667.58
2-Methoxy-4-vinylphenol	13.34±1.48	24.06±4.32	16.63±7.85	ND	18.79±4.05	29.69±4.18	20.14±0.27	11.71±20.28	23.63±2.59	78.41±10.58	72.99±13.95	125.72±24.01	26.22±1.92	83.85±16.45	124.48±13.82	92.53±17.67
1-Butanol	ND	702.91±86.91	716.95±112.17	995.72±251.59	ND	228.05±32.19	256.89±159.66	632.85±377.15	ND	574.62±270.60	1317.40±887.75	2529.76±944.09	ND	ND	258.85±93.29	234.74±43.82
1-Hexanol	3.27±0.41	18.14±8.79	27.44±24.57	56.06±36.66	8.20±2.74	34.49±4.05	38.36±25.43	180.96±249.29	6.45±0.87	47.91±39.57	38.12±13.14	110.59±44.67	ND	24.35±2.60	ND	ND
2,4-Di-tert-butylphenol	7.62±2.23	15.14±1.51	20.13±3.04	64.94±10.33	ND	ND	ND	63.37±7.68	11.23±2.31	28.91±1.92	ND	87.79±18.80	11.09±4.91	38.91±3.64	ND	37.21±5.10
2-Methoxy-4-methylphenol	ND	8.39±10.31	ND	ND	ND	16.68±3.61	75.89±34.62	119.80±14.29	ND	22.88±20.67	26.97±9.65	28.01±27.24	ND	17.21±16.11	99.82±27.81	91.25±44.65
4-Ethylphenol	ND	52.12±21.22	91.38±22.33	132.86±39.04	ND	ND	16.58±15.11	24.03±20.82	ND	ND	33.73±10.12	72.68±14.17	ND	ND	ND	ND
2,3-Dihydrobenzofuran	8.39±0.89	12.27±1.39	4.98±4.32	ND	11.14±1.72	18.63±4.82	ND	ND	14.99±1.48	55.90±4.12	66.39±5.72	152.39±13.20	14.96±4.07	44.19±8.43	ND	ND
Acetic acid	ND	19.76±3.17	14.58±17.34	27.52±34.92	ND	ND	ND	32.36±28.62	ND	ND	ND	ND	ND	ND	ND	26.46±23.55
Phenylacetic acid ethyl ester	ND	ND	6.57±6.55	ND	ND	19.79±0.79	29.40±2.40	34.31±3.70	ND	ND	ND	ND	ND	28.01±5.39	68.35±77.59	49.21±9.47
Hexanoic acid, methyl ester	6.21±2.58	ND	ND	ND	ND	ND	ND	ND	17.14±9.25	36.58±3.31	154.25±133.78	ND	ND	91.00±21.24	ND	ND
Butanoic acid, phenylethyl ester	ND	ND	ND	5.77±5.12	ND	ND	ND	ND	ND	26.12±9.65	81.48±39.28	275.64±64.80	ND	26.62±2.93	122.43±44.43	92.62±15.88
Heptadecanoic acid, 15-methyl-, ethyl ester	ND	ND	ND	ND	4.38±1.36	102.42±21.79	71.04±21.27	161.9±22.91	ND	132.22±11.50	150.42±6.62	344.25±35.08	ND	250.79±77.06	276.35±50.31	353.44±120.95
Decanoic acid, ethyl ester	ND	ND	ND	ND	4.33±0.52	170.41±43.93	127.45±3.09	96.41±84.84	5.23±4.59	133.06±10.23	168.04±15.63	126.61±22.10	ND	268.58±53.77	324.08±188.05	186.35±57.35
Lactic acid, ethyl ester	ND	ND	ND	ND	ND	499.39±104.90	1988.42±444.03	2820.91±847.58	ND	126.45±1.14	225.06±34.89	ND	ND	869.49±259.00	5277.32±1152.71	4868.01±708.45

Heptadecanoic acid, ethyl ester	ND	ND	ND	ND	ND	25.96±4.53	28.89±6.50	57.73±4.29	ND	15.66±0.72	ND	26.11±23.12	ND	35.50±11.04	60.05±52.15	106.76±25.91
Tetradecanoic acid, 13-methyl-, ethyl ester	ND	ND	ND	ND	2.65±0.52	86.95±27.91	72.72±10.77	94.82±43.28	ND	26.07±5.06	12.07±10.45	91.93±18.12	2.54±2.23	93.40±16.76	144.33±29.76	209.67±9.87
Pentadecanoic acid, ethyl ester	ND	ND	ND	ND	3.35±0.63	132.42±13.56	98.65±33.14	152.26±31.72	ND	63.61±13.01	47.78±0.70	137.91±119.85	ND	104.19±20.87	205.67±39.58	241.74±28.37
Acetic acid ethyl ester	ND	ND	ND	ND	11.99±7.31	751.31±38.02	585.29±122.78	752.00±124.99	ND	196.47±25.11	337±32.41	269.73±23.72	ND	831.24±177.52	1797.66±604.12	1176.58±333.90
Dodecanoic acid, ethyl ester	ND	ND	ND	ND	2.59±0.63	130.73±27.47	124.96±11.29	130.01±82.20	3.03±0.93	115.95±6.30	104.28±7.55	248.75±109.89	1.22±2.12	213.11±51.63	475.91±227.91	279.98±38.27
Eicosane	ND	ND	ND	ND	ND	34.39±9.08	ND	ND	ND	171±7.23	263.99±91.21	1030.79±772.53	ND	63.21±16.30	2253.51±2324.50	454.56±81.69
(Z)-Ethyl heptadec-9-enoate	ND	ND	ND	ND	ND	ND	21.44±11.12	74.43±7.06	ND	ND	ND	23.48±20.58	ND	ND	ND	57.85±50.11
(Z)-Ethyl pentadec-9-enoate	ND	ND	ND	ND	ND	27.81±3.16	29.11±5.32	52.42±13.93	ND	15.29±0.46	16.26±14.08	72.89±11.46	ND	34.75±7.80	79.58±17.84	95.79±24.89
3-Phenylpropionic acid ethyl ester	ND	ND	ND	ND	ND	66.09±29.19	83.20±32.66	152.50±58.05	ND	44.01±6.16	82.60±11.45	186.98±5.85	ND	76.09±4.29	344.93±147.76	176.46±19.76
9,12,15-Octadecatrienoic acid, ethyl ester	ND	ND	ND	ND	ND	148.01±36.78	109.57±32.22	233.66±64.97	ND	81.68±5.68	116.61±2.70	206.04±18.42	ND	144.07±34.65	245.38±22.77	262.29±109.03
9,12-Octadecadienoate, butyl ester	ND	ND	ND	ND	ND	ND	12.29±3.55	28.49±25.36	ND	ND	30.95±11.45	62.80±1.98	ND	ND	ND	29.19±26.41
9,12-Octadecadienoate, ethyl ester	ND	ND	ND	ND	ND	60.40±14.53	66.65±22.44	84.40±29.49	27.48±1.20	100.35±59.58	127.1±30.51	95.88±83.09	4.93±4.29	107.89±29.39	159.83±24.80	155.56±155.51
Butanedioic acid, diethyl ester	ND	ND	ND	ND	ND	44.22±8.53	67.23±5.23	78.49±9.89	ND	33.64±3.57	109.09±19.78	178.25±42.69	ND	202.25±25.84	511.49±144.37	492.19±141.24
Butanoic acid, propyl ester	ND	ND	7.01±0.14	10.2±8.90	ND	ND	ND	ND	ND	ND	ND	176.95±27.04	ND	ND	ND	ND
Nonanoic acid, ethyl ester	ND	ND	ND	ND	3.15±0.68	ND	ND	ND	3.62±1.37	26.53±2.72	ND	ND	ND	ND	ND	ND
2,3-Butanediol	ND	ND	ND	ND	25.79±7.23	22.79±8.35	ND	119.06±103.73	ND	ND	ND	ND	10.96±9.8	34.90±6.11	70.05±34.12	79.23±29.25
4-Ethyl-2-methoxyphenol	ND	ND	ND	ND	ND	132.01±5.23	112.34±11.71	134.71±29.92	ND	ND	ND	ND	ND	ND	ND	65.60±14.27
Tetramethylpyrazine	ND	ND	ND	ND	111.57±24.21	76.82±5.40	32.66±6.44	21.88±1.32	ND	ND	ND	ND	45.69±40.42	91.85±29.87	57.92±52.60	ND
Pentanoic acid, 2-hydroxy-4-methyl-,ethyl ester	ND	ND	ND	ND	ND	311.37±26.49	679.22±19.23	837.77±103.81	ND	ND	ND	ND	ND	154.09±41.34	1092.35±263.19	1179.23±196.19
9,12-	ND	ND	ND	ND	ND	17.17±	12.96±	46.45±	ND	ND	ND	ND	ND	ND	ND	32.86±

Hexadecadienoate, ethyl ester						3.48	3.13	4.19								5.84
Lactic acid, butyl ester	ND	ND	ND	ND	ND	ND	110.31± 65.47	240.82± 60.72	ND	ND	ND	ND	ND	ND	50.21± 45.96	66.56± 13.37
Lactic acid isopentyl ester	ND	ND	ND	ND	ND	19.19± 1.10	106.79± 15.94	91.36± 19.09	ND	ND	ND	ND	ND	59.50± 15.44	321.86± 148.29	235.30± 42.15
Acetic acid isoamyl ester	ND	ND	ND	ND	ND	49.75± 5.65	26.12± 2.48	ND	ND	ND	ND	ND	ND	72.52± 7.95	91.78± 10.07	72.45± 26.02
2,6,10,14-Tetramethylhexadecane	ND	ND	ND	ND	ND	ND	ND	ND	ND	22.51± 0.69	67.37± 28.06	362.66± 332.79	ND	ND	555.07± 565.81	158.57± 31.05
Phenol	ND	7.51±1.82	7.58±2.87	12.99± 6.19	ND	ND	ND	ND	ND	ND	ND	ND	ND	ND	ND	ND
(E)-9-Hexadecen-1-ol	ND	10.40± 2.82	17.89± 5.75	59.01± 15.93	ND	ND	ND	ND	ND	ND	ND	ND	ND	ND	ND	ND
Dodecane	4.87±1.02	ND	12.30± 1.48	ND	ND	ND	ND	ND	ND	ND	ND	ND	ND	ND	ND	ND
Pentanoic acid, butyl ester	ND	ND	5.95±5.23	36.54± 8.36	ND	ND	ND	ND	ND	ND	ND	ND	ND	ND	ND	ND
Acetic acid, butyl ester	ND	40.13± 12.50	52.04± 16.96	84.95± 28.25	ND	ND	ND	ND	ND	ND	ND	ND	ND	ND	ND	ND
Benzyl alcohol	ND	ND	ND	ND	3.05±0.63	18.87± 2.74	13.01± 0.96	ND	ND	ND	ND	ND	ND	ND	ND	ND
2,3,5-Trimethylpyrazine	ND	ND	ND	ND	16.29± 2.77	14.43± 1.22	ND	ND	ND	ND	ND	ND	ND	ND	ND	ND
(E)-2-Octenal	ND	ND	ND	ND	1.59±1.41	15.51± 1.57	ND	ND	ND	ND	ND	ND	ND	ND	ND	ND
6,9,12-Hexadecatrienoic, ethyl ester	ND	ND	ND	ND	ND	45.76± 6.96	37.07± 10.96	ND	ND	ND	ND	ND	ND	ND	ND	ND
2-Hydroxypropionic acid isobutyl ester	ND	ND	ND	ND	ND	28.32± 3.88	16.53± 1.69	ND	ND	ND	ND	ND	ND	ND	ND	ND
Propanoic acid	ND	ND	ND	ND	ND	ND	ND	ND	ND	33.55± 1.37	38.00± 7.74	ND	ND	ND	ND	ND
Butanoic acid, 2-methylpropyl ester	ND	ND	ND	ND	ND	ND	ND	ND	ND	ND	ND	98.31± 13.36	ND	ND	ND	ND
Isobutyricacid, pentyl ester	ND	ND	ND	ND	ND	ND	ND	ND	ND	78.32± 15.97	91.69± 30.42	ND	ND	ND	ND	ND
Nonanedioic acid,1,9-diethyl ester	ND	ND	ND	ND	ND	ND	ND	ND	ND	ND	ND	ND	ND	28.47± 9.86	ND	21.70± 18.79

[a] ND means not detected.
